# Do Crowding-Out Effects Explain the Low Effect of a Health Promotion Intervention among Young People at a Vocational School?

**DOI:** 10.3390/ijerph182111127

**Published:** 2021-10-22

**Authors:** Bent E. Mikkelsen, Annette Q. Romani, Maria P. Brandão

**Affiliations:** 1 Department of Geosciences and Natural Resource Management, University of Copenhagen, Rolighedsvej 23, DK-1958 Frederiksberg, Denmark; bemi@ign.ku.dk; 2 Department of Sociology and Social Work, Aalborg University, Fibigerstræde 13, 122, DK-9220 Aalborg, Denmark; aqr@socsci.aau.dk; 3 School of Health, University of Aveiro, Edifício 30, Agras do Crasto-Campus Universitário de Santiago, 3810-193 Aveiro, Portugal; 4 Center for Health Technology and Services Research, University of Aveiro, Campus Universitário de Santiago, 3800-193 Aveiro, Portugal

**Keywords:** school-based intervention, vocational education, crowding out

## Abstract

In recent years, school-based interventions have increasingly been used as a strategy to promote good eating habits and physical activity among young people at school. However, little is known about the effect that this kind of public involvement has on the overall behavior of young people. Economists refer to the existence of a crowding-out effect when public sector engagement in influencing behavior is counteracted by behaviors at the individual level. The aim of this study was to investigate the effects of a health promotion intervention program among young people at a vocational school on the overall behavior of the students and consider whether a crowding-out effect existed when it came to health behavior. This study used data from the Gearing up the Body (GUB) intervention that was carried out at the vocational school of Uddannelsescenter Holstebro, Denmark. The study included 130 students from two vocational programs. Answers were collected from survey questions in three waves. Our results showed that intervening in the school setting had the intended impact on physical activity but an unintended impact on eating behavior. In the GUB study, we found signs of countervailing behaviors in and out of school that need to be further explored.

## 1. Introduction

The increase in overweight and obesity among children and adolescents has raised worldwide attention over the past few decades. The causes of obesity and overweight are numerous but social influences are believed to play an important role. According to the socioecological model [1,2], health behavior is shaped by the different layers in our social environment. In particular, the influential role of schools attracts much attention since schools are centers of learning and education and affect the behavior of young people in multiple ways. In schools, it is possible to intervene both in the food services of the school and the classroom through educational interventions. Obesity and health behavior, particularly among young people, have increasingly been politicized and fuelled public involvement in settings such as schools [3,4,5,6]. Since the introduction of the settings-based approach, school-based interventions have increased in popularity [7,8,9]. This is not only the case for primary and secondary school. Most recently, post-secondary education has been the target of health promotion interventions [5,10], in particular, because inequalities in health seem to become much more obvious in this age group. Food-focused interventions in primary and secondary education are densely covered in the literature. Reviews over the past decade cover more than 150 studies [5,11,12,13,14,15]. However, unlike the case for elementary school, health promotion at higher educational levels, such as colleges and vocational schools, has only been studied to a limited extent.

Vocational school students tend to suffer from stigmatization, as they are a group of students where an unhealthy lifestyle, overweight, and obesity are pronounced [16]. They can be disadvantaged and stigmatized in the sense that vocational schools in many contexts are associated with low status [17,18]. Furthermore, at this educational level, health behaviors seem to increasingly cluster into unhealthy and healthy groups. Studies showed that students at vocational schools do not reach an adequate level of daily physical activity [19], nor do they consume a diet that meets the nutritional recommendations. Therefore, interventions that particularly address health in a disadvantaged educational setting have been developed to be a widely chosen option.

The prevalence of overweight children and adolescents at ages 6–19 years rose from approximately 6 to 16%, a rate that held steady from 2010 onward in the United States [20,21,22]. Hence, the proportion of obese children and adolescents almost tripled over two decades. In Denmark, the same pattern was found for overweight, whereas for obesity, the numbers are far more modest. From 1980 till 2000, the percentage of obese children and adolescents aged 6–19 years rose from approximately 0.5 to 3%, a percentage that has been constant from 2010 onward [23,24].

Nevertheless, the overall health status of young people is not only about their behavior during school hours but about the totality of the influences that makes up student life. Several studies suggest that school-based interventions can be effective in improving health behavior during school hours, while it seems to be more dubious whether this increase has a beneficial spillover effect on out-of-school health behavior or whether an increase in health behavior in school implies a crowding out of school health behavior [25,26], where the crowding-out effect is the idea that the actions that are taken by the welfare state in the public sphere, for instance, at school, tend to be counteracted by what happens outside school. Crowding out in economic and fiscal policies suggests a kind of equilibrium in which the “total sum” stays the same [27,28], regardless of the public intervention in each setting. In popular culture, the idea has its parallel in the saying that “the sum of vices is constant”. However, health promotion at higher educational levels, such as colleges and vocational schools, has only been studied to a limited extent.

In the context of vocational schools, this study had two objectives: first, to examine whether a crowding-out effect in regard to in-school healthy lifestyle interventions existed and, second, if such an effect existed, whether it might have some unintended impact on students’ behavior in the sense that beneficial effects in school are outweighed by the negative effects outside school.

### Conceptual Foundation

Following the economic human production model, by including the opportunity cost of time, cost, and benefit of inputs for health behavior, one can approach the question of whether health behaviors are substitutes or complements for academic outcomes and out-of-school activities. In other words, a crucial question considering the role of school in obesity prevention effect is the degree to which children and adolescents engage in counteracting or reinforcing their behavior. On the one hand, accountability pressure implies that schools have incentives to improve test score outcomes without necessarily having to invest more in schools. As children’s and adolescents’ health is an outcome upon which schools are not held accountable, schools may allocate their resources in a way that have unintended consequences for children’s and adolescents’ health, implying a cut back on recess, physical education (PE), extracurricular sports programs, and serving beneficial food [29,30]. Hence, earlier research showed that school accountability pressures tend to have a negative and significant effect on PE levels and obesity increases [30]. On the other hand, increasing health behavior may have a positive spill-over effect on academic outcomes as health behavior was linked to the enhancement of brain function and cognition and thereby has a beneficial impact on academic performance [31]. In the same vein, motivating schoolchildren to behave healthier by increasing mandatory PE or serving healthy food might have the side effect that they adjust their health behavior out of school. For example, several studies confirmed that variations in PE curricula can increase students’ PE [32]. Yet, there is little evidence that PE reduces schoolchildren’s weight or their risk of overweight and obesity [25]. Nevertheless, PE may have a beneficial impact by making children more active, as children do not self-select into out-of-school PE. Considering food consumption, one could likewise expect these interventions to either increase or reduce the health gap. One can formulate this in- and out-of-school trade-off for vocational school students following the model of McInness and Shingle [33]. This simple model indicates how in-school policy interventions can affect students’ out-of-school health behavior:

U(H(I,O),I,O); T = T_I_ + T_O_; E = K_I_ + K_O_; C = p_I_X_I_ + p_O_X_O_

Here, students derive utility from health H and utility or disutility from health behavior in school I (subscript), as well as health behavior outside of school O, where health depends on the health behavior both in and out of school. Students display health behavior by combining time T and energy E, where time depends on the duration and energy depends on the intensity K. Further, the cost of health behavior in school is carried by the public, while the cost of health behavior out of school is privately financed. Health behavior out of school may be a complement or substitute for health behavior in school. For example, a schoolchild may substitute their health behavior in school for their health behavior out of school if unhealthy behavior in school becomes very stigmatized; alternatively, a student may complement their health behavior in school, changing their habits and thereby increasing the efficiency of health behavior out of school. Hence, students maximize their utility subject to time and energy constraints, contributing to the Lagrangian function.

Cost–benefit analysis is important regarding evaluating the impact of increasing in-school health behavior. If students are capturing some of the benefits of increasing in-school health behavior for themselves with countervailing behavior out of school, then this is an important health benefit that was ignored in most previous calculations. This would suggest that much of the existing research underestimated the benefit of greater school resources because a part of the benefit was diverted by the students. Hence, students may obtain the same health level despite an increase in school health behavior, but they are left with more leisure time, which can be used for studying. Of course, if we are focusing narrowly on the benefits to the students in school, then the existing research is informative. The implicit assumption has been, and this is true of almost all empirical studies to date, that out-of-school behavior is unaffected by school resources and is exogenous [34,35].

In sum, the current study was designed to answer the following questions: (i) What happens to a stigmatized student’s compensation behavior (crowding-out effect) when we consider in- and out-of-school PE? (ii) What happens to the compensation behavior (crowding-out effect) when we consider in- and out-of-school food consumption?

## 2. Materials and Methods

### 2.1. Procedures

In Table 1, we can observe the characteristics of participants in this study. In the first wave, 78 students participated in the survey (93.25% male), most of which were aged between 15 and 19 years old (70.51%); in the second wave, 41 of the students from the first wave participated and 13 new students participated; in the third wave, 16 students from the second wave, 14 from the first wave, and 39 new students participated.

This study used data from the Gearing up the Body (GUB) intervention [18,36].

The program targeted students at the vocational school of Uddannelsescenter Holstebro (UCH) in the city of Holstebro, Denmark. The target group was students at two educational programs of the school: the transport/logistics and auto mechanics programs. The programs were based on a whole-school approach and consisted of 3 intervention components. These were addressing students’ eating and diet, exercise and physical activity, and smoking habits.

The GUB program was initiated in August 2014 and lasted for 10 months [33]. The survey data were collected before the interventions took place in August 2014 and recollected after 5 months in December 2014 and again after 10 months in May 2015.

Food consumption in school was addressed via the “nudging in cafeteria” intervention, which was supposed to change the students’ eating habits. The intervention addressed the students’ eating habits in the cafeteria through an unconscious approach that was grounded in a more structure-oriented concept of health and lifestyle. The core element of the intervention was access and availability. The nudging implied that energy drinks were blurred using self-adhesive film and therefore harder to spot. According to the structuralized approach, this intervention was expected to affect mainly the more disadvantaged students in the risk group.

PE in school was addressed by the “physical activity” intervention, which was supposed to change the students’ exercise habits. The intervention addressed the students’ exercise habits through a conscious approach that was grounded in an individualized concept of health and lifestyle. The core element of the intervention was regulation. The PE that was directed toward the auto students took place once a week, lasted for 90 min, and included team sports, such as football and baseball. In contrast, the PE that was directed toward the students in the transport/logistics program was power breaks, which took place once a day, lasted only for 10 min, and included strength exercises. All PE-related interventions were monitored activities (with the supervision of interventions). According to the individualized approach, this intervention was expected to affect mainly the more advantaged students in the risk group, as we expected that the more advantaged students that had some sports capital could easily change their exercise habits.

### 2.2. Instruments

A specific electronic structured anonymous questionnaire for students was designed to collect general data on social and demographic characteristics, diet, exercise, and smoking habits. The students were asked to fill out their health behaviors at three stages: the baseline, halfway follow-up, and end-point follow-up. At the final round of data collection, additional questions were asked and new constructs were created based on the collected data. The survey questions were repeated throughout the three waves, focusing on eating and exercise behaviors. Further, the third wave was expanded to include questions regarding each student’s general perception of their health and their willingness to change their behavior. The interview also took place in May 2015. The analyses in this study were exclusively based on the data from the third wave, including the extra survey questions [33]. More details about the survey were detailed elsewhere [18].

### 2.3. Data Analyses

#### 2.3.1. Dependent Variables—Internal and External Symbolic Violence

An important influential component is the expectations from the social environment. The idea of soft power suggests that behavior can be influenced through subtle pathways that involve the creation of norms and expectations regarding student behavior, bodily appearance, and health status, and that this norm setting is mainly created by actors of influence; as such, it is dependent on power dimension norms and existing values in the society. Symbolic violence refers to the exertion of soft power and norms of what society considers appropriate according to the existing and prevailing health discourses in society [19]. The idea further suggests that ruling powers can exert the right health and behavior as norms, which less-advantaged social groups are covertly forced to adhere to. The ideas of soft power and symbolic violence are inspired by Bourdieu [34]. Therefore, norms regarding the right bodily appearance, the right BMI, and the right health behavior are created by individuals with high symbolic and cultural capital rather than by those who are low in such capitals. As such, soft power and symbolic violence imply that certain types of stigmatization exist. For instance, certain behaviors and health-related bodily appearances are related to certain other traits of an individual.

To account for the students’ perception of soft power, we defined a dependent variable to capture symbolic violence at two levels, namely, external and internal levels. External symbolic violence implies that the students do not want to engage in the health behaviors to the same extent as the health authorities want them to and influencers experience challenges or problems when it comes to health or health behavior; in contrast, internal symbolic violence implies that the students experience and realize a gap between their perception of having to change and the pressure from outside of having to change.

To explore the idea of whether a gap exists between what students themselves experience as problems and challenges and what are the expectations from the environment, a new variable that captured external symbolic violence (ESV) was constructed. This variable was a scale that included 9 items that addressed the students’ physical and mental health. The items were highly correlated with a Cronbach’s alpha of 0.840, validating that the scale could be constructed by adding the items. When constructing this new scale variable, we obtained a variable that ranged from 0 to 22. Overall, we saw that the students were in good physical and mental health, with an average score on the scale of 7.61. Considering each item, 23.64% of the students had a headache at least once a week, 14.55% had stomach pain, 29.09% had back pain, 9.09% were depressed, 40.00% were irritable, 29.09% felt in a bad mood, 20.00% were nervous, 43.64% had a sleeping problem, and 7.27% felt dizzy. Based on these items, we first constructed a continuous variable with values ranging from 0 to 22. Second, we constructed a dummy equal to one if the value was above the 50th percentile (7.61) and zero otherwise, as seen in Appendix A, Table A1.

To capture the internal symbolic violence (ISV), we considered two separate scales, which were “need to change” and “nice to change” regarding the students’ habits. Here, “need to change” was a variable that was captured by the question “How do you evaluate your eating and exercise habits?” where the students’ answer could be one of very bad, bad, decent, good, and very good. In contrast, “nice to change” was captured by the question “Would you like to change your eating and exercise habits?” where yes was coded as one and no was coded as zero. Cronbach’s alpha for the “need to change” scale was 0.651, whereas, for the “nice to change” scale, it was 0.756. Thus, there was a strong correlation related to “nice to change”, implying that the students thought it would be nice to change both their eating and exercise habits. The same was not true for “need to change”, where there was a lack of correlation regarding the students’ need to change their eating and exercise habits. This discrepancy between the construction of the “nice to change habits” as compared to the “need to change habits” indicated the presence of an outside “pressure”. We referred to this as “the norm” in the sense that this is what was expected of students due to the existence of generally accepted norms of how to behave. We therefore first constructed a new variable capturing the difference between “need to change” and “nice to change”. This new variable ranged from −2 to 8. Second, based on this continuous variable, we constructed a dummy variable that was equal to one if it was above the 50th percentile (2.84) and equal to zero otherwise, as illustrated in Appendix A, Table A2.

#### 2.3.2. Independent Variable—Student Types

The key independent variables were used to create four different imagined student types: “compensating behavior”, “contradictive behavior”, “neglecting behavior”, and “elite behavior”. To construct these types of behaviors, we considered PE (physical activity as compared to seated behavior) and food consumption (healthy as compared to unhealthy food consumption), both in school and out of school. One indicator was physical activity in school, where this outcome was based on two items by considering the frequency and intensity of the PE. The frequency was measured in hours and minutes and intensity (low and high PE) was captured by a gradually increasing exponential function. For physical activity out of school, this outcome was based on the frequency and intensity of the PE. Here, we defined the frequency in terms of how often during the previous 12 months that PE was practiced. Intensity (low-, moderate-, and high-intensity training) was captured using a gradually increasing exponential function. This was combined with a measure that captured seated activity in school (sedentarism). The dataset contained information about the frequency (measured in hours and minutes) and we created continuous measures of seated activity in school by adding the hours and minutes into one variable. Another indicator was food consumption in school and out of school, which was a cluster of items that captured the students’ unhealthy eating habits. Out of school was an index based on 7 items that measured the frequency of eating specific food during the previous week. The item captured unhealthy food consumption, such as snacks and energy drinks. In-school food consumption was captured by using a dimension expressing whether the students thought that unhealthy food should be served in the canteen. Based on the idea of our whole-school approach intervention, we were able to categorize out-of-school PE and unhealthy food consumption as stigmatized behavior, whereas out-of-school seated activity and healthy food consumption were categorized into non-stigmatized behavior.

Based on this in- and out-of-school behavioral index, we created four types of behavior, as illustrated in Table 2.

The first of the four constructed behavioral types was labeled “compensating behavior”, which was a dummy variable equal to one if the students behaved healthily in school but unhealthily out of school, e.g., when increasing PE in school, students may compensate by consuming more unhealthy food in school, spending more time on seated activities, and being less physically active out of school. The second type was labeled “contradictive behavior”, which was a dummy variable equal to one if the students, despite the intervention, behaved unhealthily in school but healthily out of school, e.g., students may have been acting counterintuitively by being more inactive in school but at the same time, spent less time on seated activities and being more physically active out of school. The compensating behavior capture a crowding-out effect. The third type was labeled “neglecting behavior”, which was a dummy variable equal to one if the students responded to the “whole-school approach” by behaving unhealthily both in and out of school, e.g., students may have been ignoring the intervention by being less active and consuming more unhealthy food in school, and at the same time, spent more time on seated activity and being less physically active out of school. A final type was labeled “elite behavior”, which was a dummy variable equal to one if the students behaved healthily both in and out of school, e.g., students responded to the intervention by being more active and consuming less unhealthy food in school, and at the same time, spent less time on seated activities and being more physically active out of school.

#### 2.3.3. Subgroup Analyses—Stigmatized and Non-Stigmatized Behavior

The descriptions of in-school and out-of-school behavior of the students according to stigma can be observed in Table 3 and Appendix A, Table A3. We divided the different behaviors into stigmatizing and non-stigmatizing behaviors. We expected that an “elite behavior” always reduced the exposure to both ESV and ISV, whereas “neglecting behavior” always increased the exposure to both ESV and ISV. In contrast, we expected that “compensating behavior” and “contradictive behavior” indicated heterogeneous impacts depending on whether we considered stigmatizing or non-stigmatizing behavior. Specifically, we expected both types of behavior to have an increased impact on symbolic violence when considering stigmatized behavior and no impact when considering non-stigmatized behavior. To test this, we reran all the regressions for both stigmatized and non-stigmatized behaviors.

#### 2.3.4. Heterogeneous Effect

We claim that the trade-off between in-school and out-of-school behavior can arise if the behavior of students is constrained. For instance, students can be restricted through an energy constraint in the sense that students either need to increase their intake of energy or find ways to reduce their energy expenditure if their energy demands increase. Another way the students can be forced to make trade-offs is through the time constraint in the sense that when out of school, students are forced to make a trade-off between time spent on PE, sedentary activity, and sleeping. To test for such a heterogeneous impact, we performed a rerun of the regression for students that were respectively energy and time constrained.

### 2.4. Ethical Considerations

This study was in compliance with ethical standards. Before the data collection, all participants were informed about the objectives of the study and had read and signed a form recognizing their free and informed consent.

## 3. Results

Table 4 shows the descriptive statistics that were based on *t*-tests capturing the percentage of students that were subjected to ESV and ISV by considering both stigmatized and non-stigmatized behavior. Panel A presents the trade-off between in- and out-of-school PE and panel B presents the trade-off between in- and out-of-school food consumption. The rows in the tables represent a regression model that captured the relative impact of compensation, contradictive, and neglecting behavior, respectively, as compared to elite behavior. Considering the trade-off between in- and out-of-school PE (panel A), the results indicated that for students that adopted compensating behavior, there was a statistically significant difference between the students that experienced and did not experience ISV. In contrast, considering the trade-off between in- and out-of-school food consumption (panel B), the results indicated that for students that adopted compensating behavior, there was a statistically significant difference between students that experienced and did not experience ESV. The results remained the same regardless of whether we considered stigmatized or non-stigmatized behavior. Thus, our descriptive results indicated that compensation behavior (crowding-out effect) was present for students that were subject to symbolic violence.

Table 5 presents the results of two different regressions that captured the trade-off considering health behavior in school and out of school by considering the impacts of both ESV and ISV. Panel A presents the trade-off between in- and out-of-school PE and panel B presents the trade-off between in- and out-of-school food consumption. The rows in the tables represent a regression model that captured the relative impact of compensation, contradictive, and neglecting behavior, respectively, as compared to elite behavior. Columns (1) and (2) present the impact of stigmatizing behavior, whereas columns (3) and (4) present the impact of non-stigmatizing behaviors. Panel A shows the results considering the trade-off between in-school and out-of-school PE. Here, the results indicated that students who adopted an unhealthy behavior in school will, despite their behavior out of school, experienced less ISV. Considering the stigmatized behavior in columns (1) and (2), contradictive behavior reduced ISV by 1.68%. Further, considering neglecting behavior, ISV was decreased by 1.30 and 1.93%, whereas ESV was increased by 6.10% and 2.90%. However, neglecting behavior may have had no impact on symbolic violence, as the means were blurred, capturing an increase in ESV and a reduction in ISV. Panel B shows the results considering the trade-off between in-school and out-of-school food consumption. Here, the results were barely significant, except for compensating behavior that reduced ESV by 5.29 and 3.16% considering stigmatized and non-stigmatized behavior, respectively.

Thus, our regression results indicated that for stigmatized behavior, the compensation behavior (crowding-out effect) was increased when we considered in- and out-of-school PE, whereas the compensation behavior (crowding-out effect) was decreased when we considered in- and out-of-school food consumption. The result for non-stigmatized behavior was insignificant.

According to our conceptual framework, students not only consider the trade-off regarding health behavior in school and out of school but their health behavior is also constrained by time and energy. Table 6 presents the results of two different regressions capturing each of ESV and ISV. Panel A presents the energy constraint results and panel B presents the time constraint results. The rows in the tables represent a regression model that captured the relative impacts of compensation, contradictive, and neglecting behavior, respectively, as compared to elite behavior. Columns (1) and (2) captured the impact of stigmatizing behaviors, whereas columns (3) and (4) capture the impact of non-stigmatizing behaviors. The results indicated that the contradictive behavior, which captured unhealthy behavior in school but healthy behavior out of school, increased ESV. As expected, we found that stigmatized behavior increased ESV by 4.71%, as seen in column (1). This finding remained despite considering the difference between stigmatizing and non-stigmatizing behavior. However, considering non-stigmatized behavior, the results were barely significant, as expected, as seen in column (3).

Panel B shows the results regarding the time constraint. The results indicated that students who adopted an unhealthy behavior in school (contradictive and neglecting behavior) experienced less ISV. Thus, with regard to non-stigmatized behavior, regardless of the discrepancy between stigmatized and non-stigmatized behavior, contrasting behavior reduced ISV by 2.40 and 2.34%. The results considering neglecting behavior indicated that ISV was reduced by 1.64 and 2.23%, whereas ESV was increased by 4.15 and 8.79%. Thus, our results most likely showed a blurred mean impact as it covered an increase in ESV and a reduction in ISV.

Thus, considering the impact of energy and time constraints on health behavior, the results were identical regardless of whether we considered stigmatized or non-stigmatized behavior. However, regarding adopting unhealthy behavior in school, there was a discrepancy where students that were energy constrained experienced more ESV, whereas students that were time constrained experienced less ISV. Yet, the compensation behavior (crowding-out effect) was only significant when we considered the time constraint, which increased ESV for non-stigmatized behavior.

## 4. Discussion

To answer the first question regarding the effect of compensatory behavior on PE, we drew on the idea of crowding out, which suggests that intervention in a public setting might have a countervailing effect in other settings. Using this idea, we examined whether such a phenomenon might play a role in health promotion in vocational school settings. In our study, we hypothesized that an intervention’s impact may not be as beneficial as expected since the intervention influences other dimensions of child and adolescent behavior, namely, crowding-out out-of-school health behaviors.

The results from our analysis of the data from the program indicated that intervening in the school setting may have some unintended impacts. The results suggested that the compensation behavior (crowding-out effect) was increased when we considered in- and out-of-school PE, whereas the compensation behavior (crowding-out effect) was decreased when we considered in- and out-of-school food consumption.

The data also suggested that some unintended impacts appeared, which were believed to be working through the stigmatization of certain behaviors. This kind of valorization of commonly agreed preferred behaviors represents a type of exertion of soft power [18] and can be perceived by the individual as a sort of symbolic violence [37]. As a result, it can be speculated that the traditional approaches to the promotion of good health known from primary and secondary school may not work well in the settings of vocational schools in the sense that it may only reduce the gap in food consumption but not in PE.

It is worth noting that lifestyle-related disorders and unhealthy behaviors among young people have tended to be slightly skewed. This means that, depending on their socioeconomic background, young people can be grouped into those that show the recommended behaviors and those that do not [38]. Here it should be emphasized that our study investigated vocational school students, which is a group of students that, in the public opinion, have been sorted out of the academic school system and into a more practical track. According to laypeople, these programs are less prestigious compared to academic tracks. The increasing focus on accountability implies that vocational education is less attractive and has a lower status, just as the students’ practical competences are not given sufficient credit and testing has become part of the vocational schools [39]. According to some studies, vocational school students tend to suffer from stigmatization and are students where an unhealthy lifestyle, overweight, and obesity are more pronounced [35,40,41].

Our focus on the crowding-out effect was closely related to the increased public engagement in public health issues at school. Over the past few decades, the call for school accountability has grown when it comes to taking responsibility for the health and behaviors of children. The Council of Europe Resolution on healthy eating at school, the Nordic action plan on diet and physical activity [42], the European Charter on counter-acting obesity [43], the EU White Paper on diet and physical activity, the EU School Fruit Scheme [44], and the EU report on procurement for healthier schools [45] are just a few examples of the growing number of calls for school accountability when it comes to eating habits.

A study such as this obviously has some limitations. One major critique is that our results mainly reflect the core element of the interventions. The unconscious element of the intervention being directed toward food consumption versus the conscious element of PE most likely affected our results. Another major critical point was that the sample size was small and that the drop-out rate was large. However, our data sample was large enough to observe the possible existence of a crowding-out effect.

One of the strengths of this study was that it estimated the crowding-out effect directly for PE and food consumption. As far as we are aware, no other study has compared the direct effect of crowding out. However, Haerens and their collaborators [46] found as a result of their investigation that when a healthy food intervention was not integrated with out-of-school support, the effect was significantly worse [46]. There are also several studies that consider the effect of food-based interventions in an in-school setting that likewise found mixed results. Overall, there seem to be positive results regarding in-school interventions on food consumption and healthy food alternatives [7,12,13] but none of the studies compared the direct effect of crowding out. Only a few studies provided evidence on the fact that out-of-school behavior is affected by school resources [40], though none of them evaluated school investment regarding disadvantaged groups, addressing the unintended impact that is related to signaling what is acceptable and unacceptable behavior. However, most countries have adopted guidelines and recommendations for how food should be nutritionally composed in the captive foodscapes of schools [47], and similarly, recommendations for physical activity were adopted as part of school curricular plans [48,49]. In the case of Denmark, there is no mandatory school food provision program for schools and, as a result, they have played only a facilitating role in providing facilities and time for eating (mainly provided in the form of packed lunches in lunch boxes).

Regarding physical activity, in Denmark, it is mandatory at all school levels in the form of PE and is mainly regulated regarding duration [50]. However, some studies claim that the children compensate for increasing physical activity in school by reducing their physical activity outside of school without achieving any health impact [34]. In the same vein, other researchers also found that increasing PE did not affect either leisure-time physical activity or children’s health [51,52,53,54]. Others found that increasing PE had a beneficial impact on leisure-time physical activity and children’s health [55,56,57]. In a Danish context, neither the fear nor the crime rate substantially limits the freedom of movement and mobility of young people. The vocational school students that were studied in the GUB program as outlined above are usually physically active in their daily commuting. Therefore, despite not being physically active in school or in sport, they could still reach acceptable levels of physical activity [35].

Despite the mandatory regulations of EF in Denmark, the implementations of the new requirements at the local vocational school level have not been without problems. The major barriers for vocational school students regarding sport are the lack of sports skills, combined with the increasing elite focus; thus, vocational school students either have never been involved or have been sorted out of sports. It was also shown that organized sport is mainly available in the suburbs, implying that urban young are restricted in participation due to the cost of accessibility [58,59].

In the current study, we found signs of different countervailing behaviors and these need to be studied in more detail in future research. While the above results are compelling, much more work needs to be done on this important issue. Our results indicated that we need to move beyond simply considering the health behavior in school by considering the plausible costs and benefits that are experienced by the students themselves. Further, policymakers should be aware of the unintended impacts that are related to signaling what is right and wrong behavior, thereby contributing to victim blaming. We claim that policymakers should look for new approaches that include a broader cost/benefit approach.

## 5. Conclusions

Our results indicated that for stigmatizing behavior, there was a trade-off between in-school and out-of-school health behaviors. More specifically, we found that for stigmatized students, compensation behavior (crowding-out effect) was increased when we considered in- and out-of-school PE and compensation behavior (crowding-out effect) was decreased when we considered in- and out-of-school food consumption.

Regardless of the limitations, our findings add new evidence regarding why some studies find that school health interventions have no impact or even harm those that they are expected to help.

## Figures and Tables

**Table 1:** Characteristics of the student sample.

	Mean	(SD)
Gender		
Male	0.9325	(0.2495)
Age		
15–19 years	0.7051	(0.4589)
20–24 years	0.1923	(0.3966)
25–29 years	0.0512	(0.2220)
≥30 years	0.0128	(0.1132)
Educational program		
Auto mechanic	0.7368	(0.4432)

Note: SD: standard deviation.

**Table 2:** Categorization of the four constructed behaviors.

Behavior Type	Characteristic	Example
Compensating	Behave healthily in school, but unhealthily out of school.	When increasing PE in school students may compensate by consuming more on healthy food in school, spending more time on seated activities, and being less physically active out of school.
Contradictive	Behave unhealthily in school, but healthily out of school.	Students may be acting counterintuitively by being more in active in school, but at the same time spend less time on seated activities, and being more physically active out of school.
Neclecting	Behave unhealthily both in and out of school.	Students may be ignoring the intervention by being less active and consume unhealthy food in school, and at the same time, spend more time on seated activities and being less physically active out of school.
Elite	Behave healthily both in and out of school.	By being more active and consuming less unhealthy food in school, and at the same time, spend less time on seated activities and being more physically active out of school.

**Table 3:** Description of different constraints according to stigma.

Constraints	Stigma	Description
In-school and out-of-school physical activity trade-off	Stigmatizing	In-school physical activity was a dummy variable = 1 if the students scored above the 50th percentile of this variable, 0 otherwise Out-of-school physical activity was a dummy variable = 1 if the students scored above the 50th percentile of this variable, 0 otherwise
	Non-stigmatizing	In-school seated activity was a dummy variable = 1 if the students scored above the 50th percentile of this variable, 0 otherwise Out-of-school seated activity was a dummy variable = 1 if the students scored above the 50th percentile of this variable, 0 otherwise
In-school and out-of-school food consumption trade-off	Stigmatizing	In-school unhealthy food was a dummy variable = 1 if the students scored above the 50th percentile of this variable, 0 otherwise Out-of-school unhealthy was a dummy variable = 1 if the students scored above the 50th percentile of this variable, 0 otherwise
	Non-stigmatizing	In-school healthy food was a dummy variable = 1 if the students scored above the 50th percentile of this variable, 0 otherwise Out-of-school healthy was a dummy variable = 1 if the students scored above the 50th percentile of this variable, 0 otherwise
Energy constraint	Stigmatizing	In-school physical activity was a dummy variable = 1 if the students scored about the 50th percentile of this variable, 0 otherwise In-school unhealthy food consumption was one dummy variable = 1 if the students scored above the 50th percentile of this variable, 0 otherwise
	Non-stigmatizing	In-school physical activity was a dummy variable = 1 if the students scored above the 50th percentile of this variable, 0 otherwise Out-of-school seated activity was a dummy variable = 1 if the students scored above the 50th percentile of this variable, 0 otherwise
Time constraint	Stigmatizing	Out-of-school physical activity was a dummy variable = 1 if the students scored above the 50th percentile of this variable, 0 otherwise Out-of-school seated activity was a dummy variable = 1 if the students scored above the 50th percentile of this variable, 0 otherwise
	Non-stigmatizing	Out-of-school physical activity was a dummy variable = 1 if the students scored above the 50th percentile of this variable, 0 otherwise Out-of-school sleeping activity was a dummy variable = 1 if the students scored above the 50th percentile of this variable, 0 otherwise

Note: All outcomes were drawn from the third wave of the vocational school sampling.

**Table 4:** Students that were subject to ESV and ISV who displayed stigmatized and non-stigmatized behavior.

	Stigma		Non-Stigma		Stigma		Non-Stigma	
	ESV	Not ESV	ESV	Not ESV	ISV	Not ISV	ISV	Not ISV
N	34	21	34	21	36	19	36	19
A. In-school and out-of-school physical activity								
Compensating	0.264 (0.447)	0.428 (0.507)	0.235 (0.430)	0.285 (0.462)	0.472 (0.506)	0.052 *** (0.229)	0.222 (0.421)	0.315 ** (0.477)
Contradictive	0.205 (0.410)	0.095 (0.300)	0.235 (0.430)	0.380 (0.497)	0.138 (0.350)	0.210 (0.418)	0.305 (0.467)	0.263 (0.452)
Neglecting	0.294 (0.462)	0.142 (0.358)	0.294 (0.462)	0.095 * (0.300)	0.166 (0.377)	0.368 * (0.495)	0.166 (0.377)	0.315 (0.477)
Elite	0.235 (0.430)	0.333 (0.483)	0.235 (0.430)	0.238 (0.436)	0.222 (0.421)	0.368 (0.495)	0.305 (0.467)	0.105 * (0.315)
B. In-school and out-of-school food consumption								
Compensating	0.058 (0.238)	0.285 ** (0.462)	0.088 (0.287)	0.285 ** (0.462)	0.138 (0.350)	0.157 (0.374)	0.166 (0.377)	0.157 (0.374)
Contradictive	0.470 (0.506)	0.285 (0.462)	0.529 (0.506)	0.380 (0.497)	0.388 (0.494)	0.421 (0.4507)	0.472 (0.506)	0.473 (0.512)
Neglecting	0	0	0.382 (0.493)	0.333 (0.483)	0	0	0.361 (0.487)	0.368 (0.495)
Elite	0.470 (0.506)	0.428 (0.507)	0	0	0.472 (0.506)	0.421 (0.507)	0	0

Note: Our independent variable was the different types of students. Compensating behavior captured healthy behavior in school and unhealthy behavior out of school. Contradictive behavior captured unhealthy behavior in school and healthy behavior out of school. Neglecting behavior captured unhealthy behavior in school and unhealthy behavior out of school. Elite behavior captured healthy behavior in school and healthy behavior out of school. Standard errors are in parentheses. Panel A: In-school and out-of-school physical activity behavior were captured by in-school physical activity and out-of-school physical activity when considering stigmatized behavior and in-school seated activity and out-of-school seated activity when considering non-stigmatized behavior. Panel B: In-school and out-of-school food consumption was captured by in-school physical activity and out-of-school physical activity when considering stigmatized behavior and in–school seated activity and out-of-school seated activity when considering non-stigmatized behavior. Our dependant variable was symbolic violence. External symbolic violence (ESV) was constructed as a scale that included items that addressed the students’ physical and mental health, whereas internal symbolic violence (ISV) addressed the discrepancy between the students’ “need to change” and “nice to change” regarding their behavior. * *p* < 0.1, ** *p* < 0.05, *** *p* < 0.01.

**Table 5:** The impacts of ESV and ISV on different types of students.

	Stigma		Non-Stigma	
	ESV	ISV	ESV	ISV
A. In-school and out-of-school physical activity				
Compensating	2433 (2219)	1620 *** (0.661)	−1266 (2.625)	−0.069 (0.877)
Contradictive	2100 (3215)	−1679 ** (0.861)	−0.743 (2478)	−0.300 (0.863)
Neglecting	6100 *** (2431)	−1300 * (0.715)	2904 (2879)	−1925 ** (0.989)
B. In-school and out-of-school food consumption				
Compensating	−5292 ** (2402)	−0.311 (0.933)	−3166 (2600)	0
Contradictive	−1900 (1997)	−1022 (0.690)	−0.311 (2100)	−0.220 (0.903)
Neglecting	0	0	0	−0.500 (0.941)

Note: Our independent variable was the different types of students. Compensating behavior captured healthy behavior in school and unhealthy behavior out of school. Contradictive behavior captured unhealthy behavior in school and healthy behavior out of school. Neglecting behavior captured unhealthy behavior in school and unhealthy behavior out of school. Elite behavior captured healthy behavior in school and healthy behavior out of school. Panel A: In-school and out-of-school physical activity behavior were captured by in-school physical activity and out-of-school physical activity when considering stigmatized behavior and in-school seated activity and out-of-school seated activity when considering non-stigmatized behavior. Panel B: In-school and out-of-school food consumption was captured by in-school physical activity and out-of-school physical activity when considering stigmatized behavior and in-school seated activity and out-of-school seated activity when considering non-stigmatized behavior. Our dependant variable was symbolic violence. ESV was constructed as a scale that included items that addressed the students’ physical and mental health, whereas ISV addressed the discrepancy between the students’ “need to change” and “nice to change” regarding their behavior. * *p* < 0.1, ** *p* < 0.05, *** *p* < 0.01.

**Table 6:** The heterogeneous impacts of ESV and ISV on different types of students.

	Stigma		Non-Stigma	
	ESV	ISV	ESV	ISV
Panel A. Energy constraint				
Compensating	−2955 (2950)	−0.600 (0.961)	2333 (3304)	−1178 (0.990)
Contradictive	4717 ** (2377)	1400 (0.935)	4318 * (2615)	1068 (0.869)
Neglecting	0.301 (2259)	0.150 (0.843)	4285 * (2494)	0.500 (0.806)
Panel B. Time constraint				
Compensating	−1233 (2283)	0.375 (0.706)	4789 ** (2394)	−0.060 (0.862)
Contradictive	2200 (2501)	−2400 *** (0.792)	2989 (1997)	−2342 *** (0.655)
Neglecting	4150 (3309)	−1642 * (0.880)	8789 *** (2812)	−2227 ** (1017)

Note: Our independent variable was the different types of students. Compensating behavior captured healthy behavior in school and unhealthy behavior out of school. Contradictive behavior captured unhealthy behavior in school and healthy behavior out of school. Neglecting behavior captured unhealthy behavior in school and unhealthy behavior out of school. Elite behavior captured healthy behavior in school and healthy behavior out of school. Panel A: The energy constraint was captured by in-school physical activity and in-school unhealthy food consumption when considering stigmatized behavior and in-school physical activity and out-of-school seated activity when considering non-stigmatized behavior. Panel B: The time constraint was captured by out-of-school physical activity and out-of-school seated activity when considering stigmatized behavior and out-of-school physical activity and out-of-school sleeping activity when considering non-stigmatized behavior. ESV was constructed as a scale that included items that addressed the students’ physical and mental health, whereas ISV addressed the discrepancy between the students’ “need to change” and “nice to change” regarding their behavior. * *p* < 0.1, ** *p* < 0.05, *** *p* < 0.01.

## Data Availability

The datasets that were analyzed in this study are not publicly available but are available on request from the corresponding author.

## Appendix A

**Table A1:** Description of the External Symbolic Violence (ESV) Scale.

Outcome	Description	Outcome Range	Mean (SD)
Physical and mental health	Cronbach’s alpha = 0.840	0–27	7615 (5678)
Headache	Dummy variable = 1 if the student had pain at least once a week, zero otherwise	0–1	0.236 (0.428)
Stomach pain	Dummy variable = 1 if the student had pain at least once a week, zero otherwise	0–1	0.145 (0.355)
Back pain	Dummy variable = 1 if the student had pain at least once a week, zero otherwise	0–1	0.290 (0.458)
Sad	Dummy variable = 1 if the student felt sad at least once a week, zero otherwise	0–1	0.090 (0.290)
Irritable	Dummy variable = 1 if the student felt irritable at least once a week, zero otherwise	0–1	0.400 (0.490)
Bad mood	Dummy variable = 1 if the student was in a bad mood at least once a week, zero otherwise	0–1	0.290 (0.458)
Nervous	Dummy variable = 1 if the student was nervous at least once a week, zero otherwise	0–1	0.200 (0.403)
Sleeping problems	Dummy variable = 1 if the student had sleeping problems at least once a week, zero otherwise	0–1	0.436 (0.500)
Dizzy	Dummy variable = 1 if the student felt dizzy at least once a week, zero otherwise	0–1	0.072 (0.262)

**Table A2:** Description of the Internal Symbolic Violence (ISV) Scale.

Outcome	Description	Outcome Range	Mean (SD)
Need to change habits	Cronbach’s alpha = 0.651		
Eating	The possible answers were very bad, bad, decent, good, and very good	1–4	0.400 (0.494)
Exercise	The possible answers were very bad, bad, decent, good, and very good	1–4	0.309 (0.466)
Nice to change habits	Cronbach’s alpha = 0.756		
Eating	Dummy variable = 1 if the student replied yes, 0 otherwise	0–1	0.290 (0.458)
Exercise	Dummy variable = 1 if the student replied yes, 0 otherwise	0–1	0.2902 (0.4583)

**Table A3:** Description of the In-School and Out-of-School Behavior.

Outcome	Description	Outcome Range	Mean (SD)
Stigmatizing behavior			
In-school physical activity	Standardized continuous index of intensity and frequency of physical activity level based on 3 items (each measured on a 6-point scale and gradually increased as an exponential function)	0–1 (0–18)	0.5722 (0.3277)
Out-of-school physical activity	Standardized continuous index of intensity and frequency of physical activity level based on 2 items (each measured in hours and minutes and gradually increased as an exponential function)	0–1	0.5722 (0.3277)
In-school unhealthy food consumption	Standardized continuous index (derived from 7 items, each measured on a 4-point scale) that determined whether the students had healthy eating habits	0–1 (0–42)	0.4872 (0.1114)
Non-stigmatizing behavior			
In-school seated activity	Standardized continuous index of the frequency of seated activity level in school (measured in hours and minutes)	0–1	0.5722 (0.3277)
Out-of-school seated activity	Standardized continuous index of seated activity level out of school (derived from 2 items, measured on an 8-point scale)	0–1 (0–16)	0.5722 (0.3277)
Out-of-school sleep activity	Standardized continuous index of frequency of sleep out of school (measured in hours and minutes)	0–1	0.5722 (0.3277)
